# The endurance effort on a mechanical treadmill does not activate the complement system among trained young women

**DOI:** 10.1371/journal.pone.0353880

**Published:** 2026-07-16

**Authors:** Robert Nowak, Patrizia Proia, Dorota Kostrzewa-Nowak

**Affiliations:** 1 Institute of Physical Culture Sciences, University of Szczecin, Szczecin, Poland; 2 Department of Pathology, Pomeranian Medical University in Szczecin, Szczecin, Poland; 3 Sport and Exercise Sciences Research Unit, Department of Psychology, Educational Science and Human Movement, University of Palermo, Palermo, Italy; 4 Department of Clinical and Molecular Biochemistry, Pomeranian Medical University in Szczecin, Szczecin, Poland; Kansai Medical University: Kansai Ika Daigaku, Institute of Biomedical Science, JAPAN

## Abstract

The complement system (CS) plays a crucial role not only in pathogen-related immune responses but also in immunomodulatory effects associated with increased levels of damage-associated molecular patterns and tissue regeneration. The involvement of CS in response to physical exertion remains unclear. In the present study, the impact of an endurance effort on a mechanical treadmill on CS activation was investigated in healthy young premenopausal women. Twenty-eight well-trained women aged 21–26 years, playing handball or soccer, respectively, performed a progressive test on a mechanical treadmill until exhaustion. The CS component (C1q, C2, C3, C3b/iC3b, C4, C4b, C5, C5a, Factors I, H, B, adipsin, and mannose-binding lectin) concentration in blood was analyzed before, after the test, and 24 hours after the physical test using Luminex xMAP (Multi Analyte Profiling) technology. Progressive effort did not significantly affect the analyzed CS compounds. The lack of CS activation observed in the studied groups of young women suggests that the endurance effort on a mechanical treadmill did not activate an innate immunity. From this perspective, it does not appear to be an inflammatory factor. Considering the small number of participants, this hypothesis needs further exploration.

## Introduction

The complement system (CS) comprises plasma and membrane-bound proteins that play a crucial role in maintaining an organism’s defense and adaptation to various stimuli, such as bacterial or viral pathogens, physical activity, or oxidative stress [1–5]. The majority of fluid-phase proteins are produced by the liver. However, mast cells, macrophages, dendritic cells, and smooth muscle cells also contribute to both the fluid-phase and membrane-bound pool [2,6,7].

Complement system activation is widely discussed in the literature, and the post-effort response is influenced by numerous factors, including participants’ training experiences, age, sex, and the type of effort (e.g., short- or long-term, aerobic, or anaerobic). Beyond duration and metabolic character of exercise, the kind of muscle contraction, movement patterns, and atmospheric oxygen availability all play a role in the modulation of complement activation and the organism’s response [5]. Broadly, moderate aerobic training in healthy women tends to reduce complement components at rest, suggesting less systemic complement activation and possibly less chronic low-grade inflammation [8]. Chronic high-level endurance training (e.g., among elite athletes) may further reduce resting levels of certain complement factors (e.g., C2, C3), potentially reflecting adaptation to reduce unnecessary complement-mediated inflammation [9]. After intense, prolonged acute endurance effort, there may be transient consumption of complement, evidenced by a decrease in certain components, such as C3 and an increase in cleavage products, such as C3a [10].

Biological sex substantially influences CS activity. Costa et al. reported significantly lower alternative pathway (AP) activity in females than in males, with lower C3 and properdin levels and higher factor D concentration in females. Mannose-binding lectin-lectin pathway (MBL-LP) activity was not influenced by sex, but MBL and ficolin-3 levels were significantly lower in females [11]. These differences are driven largely by hormones: estrogen enhances immune activation, promoting inflammation and increasing susceptibility to autoimmune disease, while testosterone exerts primarily immunosuppressive effects, reducing autoimmune risk but increasing susceptibility to infection [12,13]. Androgens and progesterone promote immunosuppressive or immunomodulatory effects, whereas estrogen enhances humoral immunity in both males and females [13].

The most frequently studied complement fragment in the context of physical fitness and exercise is C3, followed by C4- most likely because of their utility as markers of inflammation and C3’s central role in complement activation, as all three pathways converge at the C3 cleavage step. C3 also functions as an adipokine and inflammatory marker associated with insulin resistance [5,14,15]. Serum concentration of complement fragments corresponds with the severity of low-grade inflammation associated with obesity, increased fat percentage, and low physical fitness [14–17]. In healthy women, both acute and chronic endurance exercise generally reduces baseline levels of several complement proteins (C1q, C3, C4, factor B, properdin), reflecting reduced inflammation and improved immune regulation [8]. Acute endurance activity may cause transient system activation, reflected by an increase in cleaved components such as C3a and C5a alongside decreases in total C3 [10,18], whereas elite endurance-trained women show lower levels of proinflammatory complement components at rest, indicating long-term adaptation [19].

A brief review of the impact of endurance effort on a mechanical treadmill on complement system proteins in the female population is presented in Table 1, and the comparison between males and females is presented in Table 2.

**Table 1:** Complement system activation in response to endurance exercise among the female population.

Complement Component	Function	Acute Exercise (Single Bout)	Chronic Endurance Training	Notes	Ref.
**C1q**	Classical pathway	Not reported	↓ in healthy women after training	No significant change in PCOS women	[8]
**C2**	Classical pathway	Not reported	↓ in high-endurance athletes	Associated with reduced baseline inflammation	[9]
**C3**	Central component in all pathways	↓ post-exercise (may reflect activation)	↓ after training (healthy women)	Higher at baseline in PCOS; no training-induced reduction	[8,10,20]
**C3a**	Anaphylatoxin (pro-inflammatory fragment of C3)	↑ after intense exercise	Not consistently measured	Often increases transiently after exhaustive exercise	[18,20]
**C4**	Classical and lectin pathways	↑ slightly post-fat meal	↓ with training (healthy women)	No significant change in PCOS	[8,20]
**C5**	Terminal component → forms MAC	Slight ↑ or stable post-exercise	Not clear	Elevated in some PCOS studies	[18]
**C5a**	Potent anaphylatoxin	↑ after acute exercise in some studies	Not clear	Related to muscle damage, neutrophil activation	[18]
**C3b/iC3b**	Opsonins (enhance phagocytosis)	↑ transiently post-exercise	↓ in chronic endurance athletes	Lower in high-endurance female athletes	[19]
**Properdin**	Stabilizes the alternative pathway	↓ with training (healthy women)	↓ in chronic endurance	No change in PCOS women with training	[8]
**Factor B**	Alternative pathway activator	No substantial acute change reported	↓ in healthy women after aerobic training	Elevated in PCOS; no change with training	[8,20]
**Factor D (Adipsin)**	Alternative pathway protease	Not directly studied in exercise	↓ in endurance-trained athletes	Associated with body composition and metabolism	[19]
**Factor H**	Regulator (inhibits the alternative pathway)	↑ post-fat meal in PCOS	↓ with training in healthy women	Compensatory increase in PCOS	[8,20]

MAC – membrane attack complex; POCS – polycystic ovary syndrome; Ref. - references.

**Table 2:** The comparison of complement system activation between males and females [11–13].

Feature	Females	Males
**Alternative pathway (AP) activity**	↓ Lower	↑ Higher
**C3 (baseline)**	↓ Lower	↑ Higher
**Properdin (baseline)**	↓ Lower	↑ Higher
**Factor D (baseline)**	↑ Higher	↓ Lower
**MBL / ficolin-3 (lectin pathway)**	↓ Lower	↑ Higher
**Classical pathway activity**	≈ Similar	≈ Similar
**Terminal components (C5–C9)**	↓ Lower	↑ Higher
**Complement response to aerobic exercise**	Attenuated (esp. PCOS)	Activation of AP was documented
**Sex hormone influence**	Estrogen enhances humoral immunity	Testosterone suppresses immune activation

Despite this growing body of evidence, the post-effort complements activation and regeneration kinetics following endurance effort in women remain poorly mapped. It is still unclear how quickly component cleavage products rise after exercise, how long elevated complement activity persists, and how factors such as menstrual cycle phase influence these responses. The pilot proteomic study found no significant association between cycle phase and C3 changes [10], and the mechanism by which complement regulation can be altered – including changes in clearance, generative capacity, regulatory proteins, or hormonal modulation – is incompletely understood in this population.

On the other hand, our previous study demonstrated that anaerobic and aerobic effort performed on fifty-one young physically active males aged 16 years (range 15–21 years) elicited different immune responses regarding CS: aerobic exercise induced activation of an alternative pathway, whilst anaerobic effort had little influence. C3 and C4 levels show a significant inverse correlation with the Beep test (20-m shuttle run test) results, and the C3/C4 ratio differs markedly between effort types and time points [4].

Building on these findings, the present study aimed to compare the contribution of CS to post-effort response among healthy, well-trained young women training in team sports, namely handball or soccer. General motor and sport physiology differences between those team sports are related to the involvement of strength and endurance factors during both the training and the game itself. Both handball and soccer are complex and multifactorial team sports [21,22]. The intensities during the handball game change between standing and walking, jogging and moderate running, sprinting, and rapid forward, sideward, and backward movements. This sport requires players to utilize power, strength, and a high level of endurance [23]. The VO_2_max in elite and experienced handball players during an incremental treadmill-running test is greater than 55 mL/kg/min [18], whereas among soccer players it is greater than 50 mL/kg/min [22,24]. Moreover, soccer requires a lower strength component from players compared to handball [24,25].

The research questions put in this study were: (i) Does the progressive effort until exhaustion cause an activation of CS? (ii) Which activation pathway is involved in post-effort CS activation among the studied groups? (iii) Is there a difference in the activation level or activation pathways depending on the type of sport discipline?

## Materials and methods

### Study design

**Fig 1:**
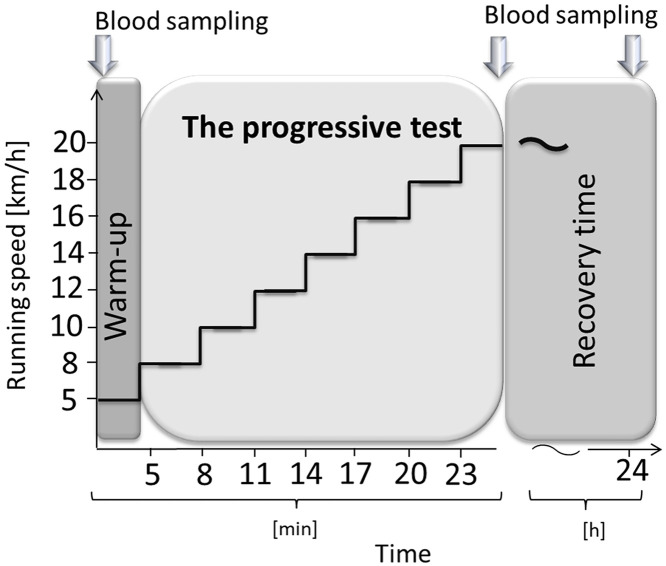
The experimental protocol of the progressive test until exhaustion and blood sampling.

The impact of an endurance effort on a mechanical treadmill on CS activation was studied among healthy young women. Professional athletes, highly qualified handball and soccer players who belong to sports clubs, were recruited for this study. All participants were asked to perform progressive test on a mechanical treadmill until exhaustion according to the protocol described previously [26] (Fig 1).

The study was conducted on the first day of the new preparatory stage, two weeks after the summer holidays, when the participants were free from regular training units. The test was performed in the morning under laboratory conditions, 2 hours after a light breakfast and following a period of two days without physical activity, at a temperature of 20–23°C. The test was preceded by a 5-minute warm-up run on a mechanical treadmill at a speed of 5 km/h. During the test, the speed increased by 2 km/h every 3 minutes until exhaustion, i.e., until the participant refused to run due to extreme fatigue. To study CS activation, venous blood samples were collected three times: before the test (before the warm-up), immediately after the test (within 5 minutes), and after a night's recovery (approximately 24 hours after the test). The recovery time was free from physical activity, including any training units. Those 24 hours were a typical rest time for the participants.

The plasma concentrations of selected complement proteins, namely C1q, C2, C3, C3b/iC3b, C4, C4b, C5, C5a, Factors I, H, B, adipsin, and mannose-binding lectin (MBL), were measured using Luminex xMAP (Multi Analyte Profiling) technology according to the manufacturer’s protocol. In addition, to compensate for the changes in cell counts induced by the exercise test, plasma volume loss (ΔPV) and the subsequent correction of those parameters for ΔPV were calculated according to the classic equations from Dill and Costill, provided by Alis et al. [27]:


$$\begin{array}{c} \Delta PV\;(\%)=\text{1}\text{0}\text{0}\times \left(\frac{{Hb}_{pre}}{{Hb}_{post}}\times \frac{\text{1}\text{0}\text{0}-{Htc}_{post}}{\text{1}\text{0}\text{0}-{Htc}_{pre}}-\text{1}\right) \end{array}$$

where: Hb_pre_  =  hemoglobin pre-test (g/dL); Hb_post_  =  hemoglobin post-test (or in recovery; g/dL); Htc_pre_  =  hematocrit pre-test (%); and Htc_post_  =  hematocrit post-test (or in recovery; %).

The formula for the correction of blood parameters was as follows:


$$\begin{array}{c} {}[Corrected\;parameter\;concentration]=[Uncorrected\;parameter\;concentration]\times \left(\text{1}+\frac{\Delta PV(\%)}{\text{1}\text{0}\text{0}}\right) \end{array}$$

The same formula was used in our previous study [4].

Hematocrit value and hemoglobin concentration were analyzed using the hematology analyzer ABX Micros 60 (Horiba ABX, Warsaw, Poland). Additionally, the level of inflammatory markers was calculated as the mean C3/C4 plasma ratio [28].

### Participants

Twenty-eight women aged 20–29 years old were recruited for this study (between 01. Dec. 2016 and 01. Jul. 2017).

There were 14 handball players aged 20–29 years old, and 14 soccer players aged 20–26 years old. Before the warm-up, the participant’s body mass and body composition parameters, namely body mass index, basal metabolic rate, percentage of fat, fat-free mass, and total body water, were determined using a body composition analyzer (Tanita BC-418MA; Tanita, Tokyo, Japan). Maximal oxygen consumption (VO_2_max) was determined using a gas exchange data analyzer Quark CPET (Cosmed, Albano Laziale, Italy).

The main inclusion criteria included sex, practicing an appropriate sport discipline, being a non-smoker, and refraining from taking any medications or supplements known to affect metabolism. Moreover, the participants had to have no history of any metabolic syndrome or cardiovascular diseases. Additionally, they had no medically detected hormonal disorders or immune system failure. Participants who did not meet abovementioned inclusion criteria or who did not give or withdraw their consent to participate were excluded from the study.

All procedures were conducted in accordance with the ethical standards outlined in the Declaration of Helsinki. The research was approved by the Bioethical Commission at the Local Branch of the Medical Chamber in Szczecin (approval No. 13/KB/V/2014). Written informed consent was obtained from all subjects before taking part in the study.

### Blood sampling

Venous blood was drawn from the elbow vein in accordance with standard diagnostic procedures at the three time points described above. Each time, blood samples were taken into 7.5 mL S-Monovette tubes with ethylenediaminetetraacetic acid (K3EDTA, 1.6 mg EDTA/mL blood) (SARSTEDT AG & Co., Nümbrecht, Germany). Blood samples were centrifuged at 2000 × g for 10 minutes at room temperature. The blood plasma was collected for future analysis.

### Biochemical analysis

Albumin, total protein (TP), C-reactive protein (CRP), and lactate (LA) concentrations and creatine kinase (CK) activity were determined using a colorimetric assay kit (BioMaxima S.A., Lublin, Poland for albumin, TP, CRP and PZ Cormay S.A., Łomianki, Poland for LA, respectively) according to the manufacturer’s protocol with the use of an Automatic Clinical Chemistry Analyzer (BM-100, BioMaxima S.A., Lublin, Poland). All analyses were verified using a multiparametric control serum and two control sera of normal level (BioNorm) and high level (BioPath) (BioMaxima S.A., Lublin, Poland).

The baseline plasma hormone profile (follicle-stimulating hormone (FSH), luteinizing hormone (LH), progesterone (PRL), estradiol (ES)) of the participants was determined using enzyme-linked immunosorbent assays (ELISAs) according to the manufacturer’s protocol (DRG MedTek, Warsaw, Poland). All ELISA tests were performed using a high-throughput microplate reader, Synergy H1 (BioTek Instruments, Inc., Vermont, USA).

### Complement System Activation Study

The plasma concentrations of all the studied proteins were measured using a Bead-Based Multiplex Assay kit and the Luminex xMAP technology (Merck KGaA, Darmstadt, Germany). MILLIPLEX® Complement Magnetic Bead Panels 1 and 2 were used for the study. Analyses were performed using MAGPIX® Luminex® Multiplexing Instruments (Merck). The concentration of the studied complement proteins was calculated using the Belays Immunoassay Curve Fitting Software (Merck).

### Statistical analysis

All data are presented as medians (range). Statistical analyses were performed using Statistica version 13 (2017; TIBCO Software Inc., Palo Alto, CA, USA; http://statistica.io). The normality of the data distribution within the subgroups was assessed using the Shapiro-Wilk test. Due to the non-normal data distribution and the small sample size, nonparametric statistical analyses were conducted. Differences between groups (handball players vs. soccer players) were assessed using the Mann-Whitney U-test. The differences between time points (pre-test vs. post-test vs. recovery) were assessed using Friedman’s analysis of variance for repeated measures followed by post-hoc Dunn tests with Bonferroni correction. For all analyses, p < 0.05 was considered significant.

## Results

The characteristics of the participants are presented in Table 2. There were no significant differences between the studied groups when examining concentrations of FSH, LH, PRL, and ES (Table 3).

**Table 3:** The characteristics of participants.

	Handball players (*n* = 14)	Soccer players (*n* = 14)
**Age (years)**	24.5 (20–29)	20 (19–21)
**Height (cm)**	174 (169–177)	181 (179–186)
**Weight (kg)**	66.7 (63.3–77.0)	76.1 (70.2–83.2)
**BMI (kg/m**^**2**^)	22.4 (22.0–24.4)	23.3 (21.6–24.4)
**BMR (kJ)**	8632 (7966–9037)	8447 (7914–8855)
**Fat (%)**	11.5 (7.9–12.3)	11.2 (8.2–13.2)
**Fat mass (kg)**	8.2 (5.6–9.9)	8.6 (5.4–10.9)
**FFM (kg)**	66.6 (62.3–68.5)	68.3 (64.5–70.9)
**TBW (kg)**	48.8 (45.6–50.1)	50.0 (47.2–51.9)
**FSH (ng/mL)**	0.57 (0.48–0.78)	0.35 (0.23–0.56)
**LH (mlU/mL)**	7.45 (4.46–18.21)	8.69 (5.11–11.14)
**PRL (ng/mL)**	1.28 (1.09–1.75)	1.17 (0.87–1.44)
**ES (pg/mL)**	78.8 (47.7–111.1)	63.5 (43.9- 79.4)
**VO**_**2**_ **max (mL/kg/min)**	50.4 (47.5–51.2)	42.5 (41.3–45.2)

The table presents the median (interquartile range), except for age, which is presented as median (minimum–maximum range) values characterizing the participants. BMI – body mass index, BMR – basal metabolic rate, ES – estradiol, FSH – follicle-stimulating hormone, LH – luteinizing hormone, PRL – progesterone, FFM – fat-free mass, TBW – total body water, VO_2_max - maximal oxygen consumption, *n* – number of participants.

The corrected values of CK activity were significantly higher at the post-test time point than at baseline (pre-test) among handball players and during recovery among soccer players (Table 4).

**Table 4:** Corrected albumin, TP, CRP, LA, and CK levels among the studied participants.

Variable		Handball players (*n* = 14)	Soccer players (*n* = 14)	p_MW_^2^
**Corrected** **albumin (g/L)**	pF ^1^	0.0015	0.0000	
	pre-test	46.8 (46.3–48.9)^aaa^	51.4 (49.7–52.7)	0.000025
	post-test	49.1 (47.2–49.3)	51.6 (51.1–53.0)	0.000005
	recovery	48.4 (47.2–49.7)^c^	49.6 (49.0–51.8)	0.027396
**Corrected TP (g/L)**	pF	0.00150	0.00544	
	pre-test	68.1 (66.9–70.5)^aaa^	71.5 (70.1–73.1)	0.002486
	post-test	71.5 (68.6–73.1)	72.4 (71.3–76.8)^bb^	0.103523
	recovery	70.0 (66.2–70.8)	68.3 (67.1–69.6)	0.482401
**Corrected CRP (mg/L)**	pF	0.00141	0.00079	
	pre-test	0.75 (0.60–1.00)	0.45 (0.00–1.20)^aa^	0.076664
	post-test	0.59 (0.20–0.98)^b^	1.22 (0.29–1.84)	0.193579
	recovery	0.00 (0.00–0.40)^cc^	0.90 (0.00–2.40)	0.024124
**Corrected LA (mmol/L)**	pF	0.00002	0.00001	
	pre-test	1.1 (0.9–1.2)^aaa^	0.5 (0.5–0.6)^aaaa^	0.000101
	post-test	12.5 (9.8–14.5)^bbb^	6.1 (4.4–7.3)^bb^	0.000001
	recovery	1.1 (0.8–1.2)	0.7 (0.6–0.8)	0.000019
**Corrected CK activity (U/L)**	pF	0.02437	0.01111	
	pre-test	229 (187–358)^a^	116 (99–130)	0.000019
	post-test	241 (198–376)	120 (108–144)	0.000034
	recovery	223 (163–391)	135 (93–158)^c^	0.000078

^1^Significance levels of differences observed between analyzed time points (pre-test vs. post-test vs. recovery) were assessed using Friedman’s analysis of variance for repeated measures (pF - Friedman’s ANOVA p-values) followed by Dunn’s post hoc test with Bonferroni correction. The table presents the median (interquartile range) of values corrected for ΔPV. TP – total protein, CK – creatine kinase, CRP – C-reactive protein, LA – lactate, n- number of participants. The analyses were performed before (baseline, pre-test) and after the effort (5 minutes post-effort and during recovery, approximately 24 h after the test). Post-hoc p values: ^a^ p < 0.05, ^aa^ p < 0.01, ^aaa^ p < 0.001, ^aaaa^ p < 0.0001, for pre-test vs. post-test, ^b^ p < 0.05, ^bb^ p < 0.01, ^bbb^ p < 0.001, for post-test vs. recovery, ^c^ p < 0.05, ^cc^ p < 0.01, for pre-test vs. recovery. ^2^ Differences between groups (handball players vs. soccer players) were assessed using the Mann-Whitney U-test.

It was found that corrected albumin and TP concentrations following endurance effort on a mechanical treadmill were higher only in the handball player group. Interestingly, recovery values of TP were similar to baseline in both studied groups, in contrast to albumin concentration, which was lower at this time point among handball players in comparison to the post-effort time point (Table 4). Conversely, CRP concentration was higher in post-effort time points compared to baseline values only in soccer players. At the same time, the CRP level was significantly lower at recovery time points than at baseline in handball players group (Table 4). Although lactate concentrations differed between handball and soccer players, a similar pattern of changes in this analyte was observed: a statistically significant increase immediately after exercise and a return to baseline values during restitution (Table 4).

**Fig 2:**
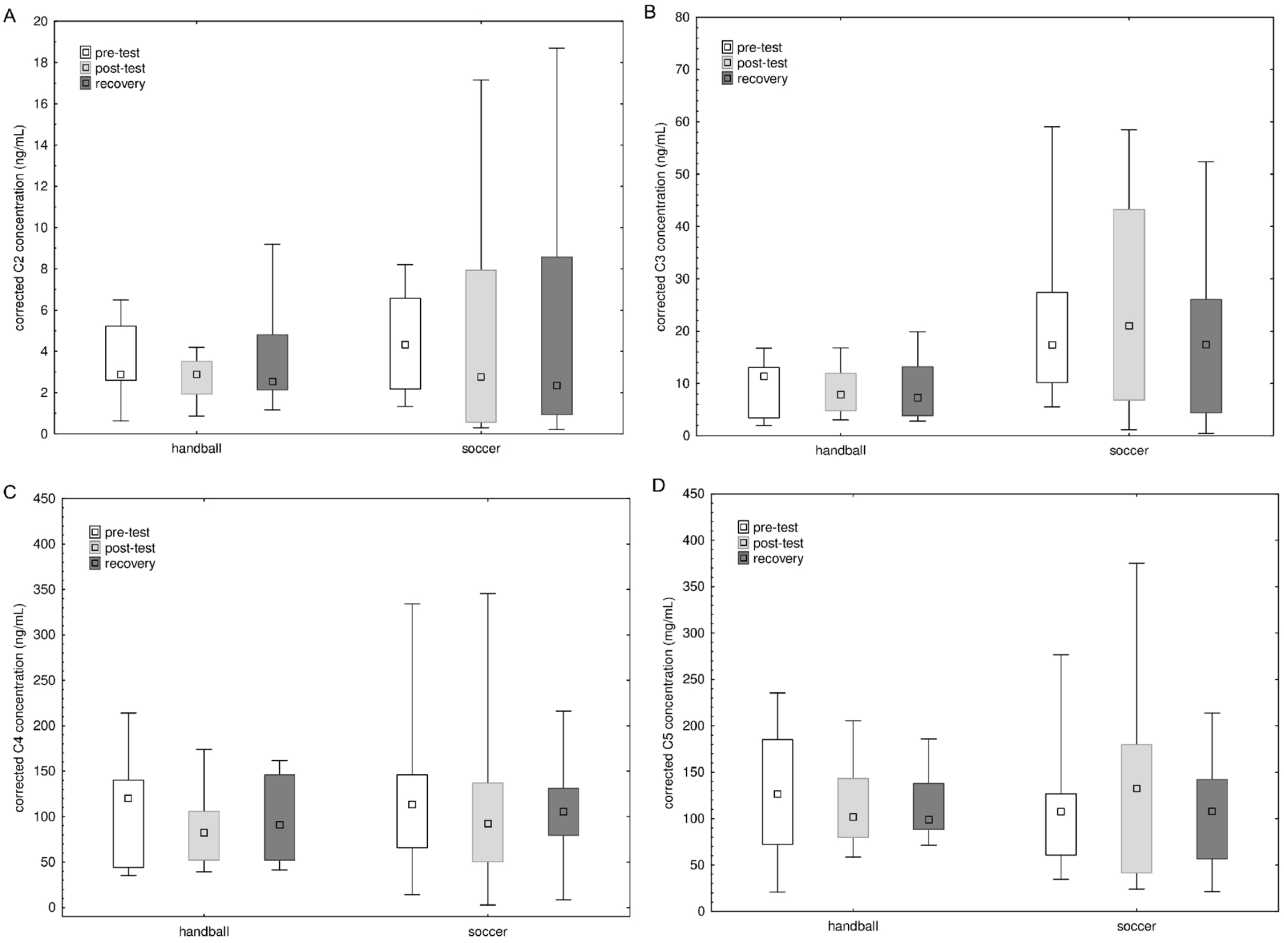
The plasma concentrations of inactive CS compounds: A) C2, B) C3, C) C4, D) C5.

**Fig 3:**
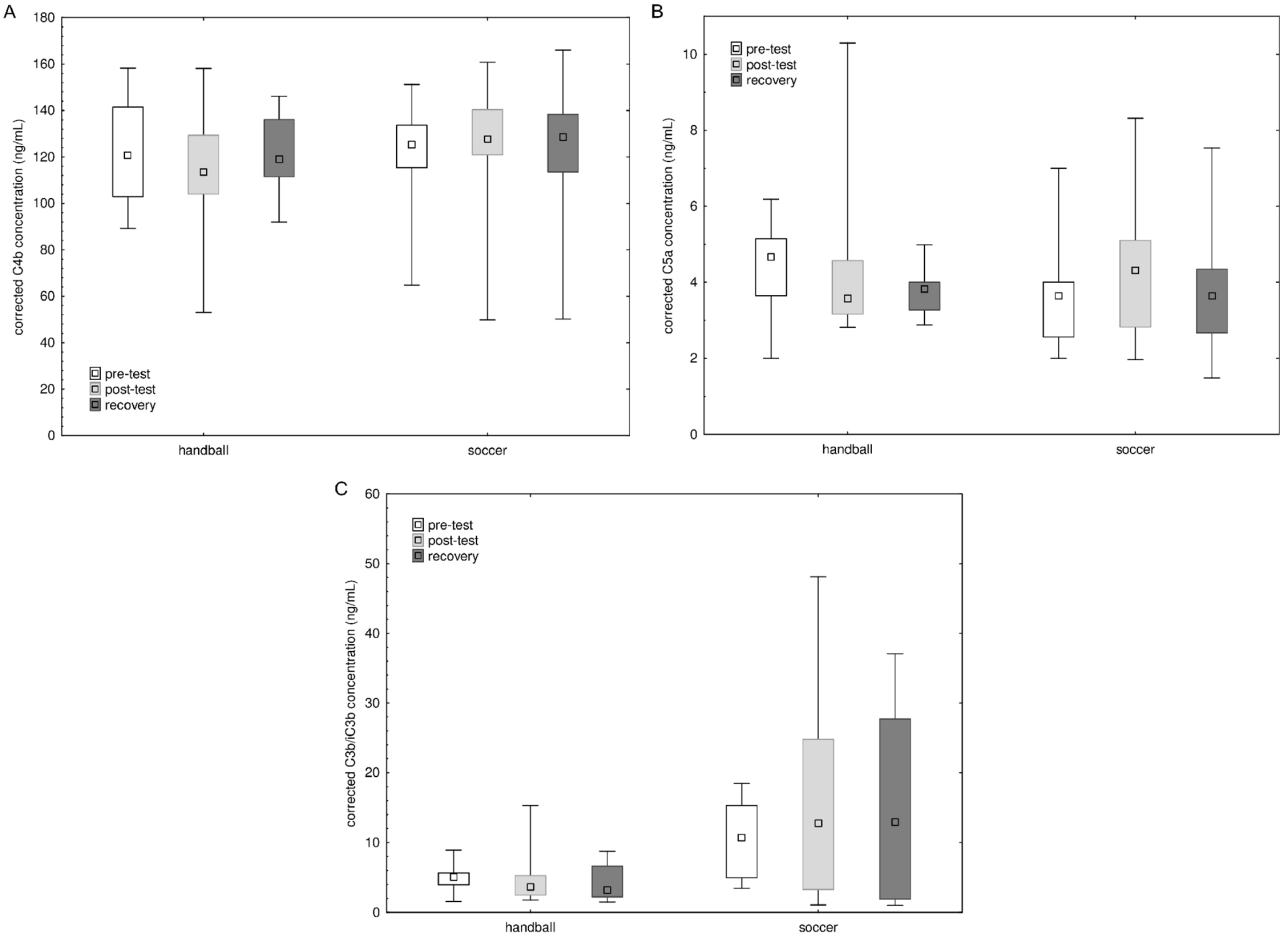
The plasma concentrations of cleaved (activated) CS compounds: A) C4b, B) C5a, C) C3b/iC3b.

It was found that after progressive effort on a mechanical treadmill, there were no changes in the corrected concentrations of inactive complement compounds (C2, C5, C3, C4) or their cleaved forms (C4b, C5a, C3b/iC3b) in either of the studied groups (Figs 2 and 3, respectively).

**Fig 4:**
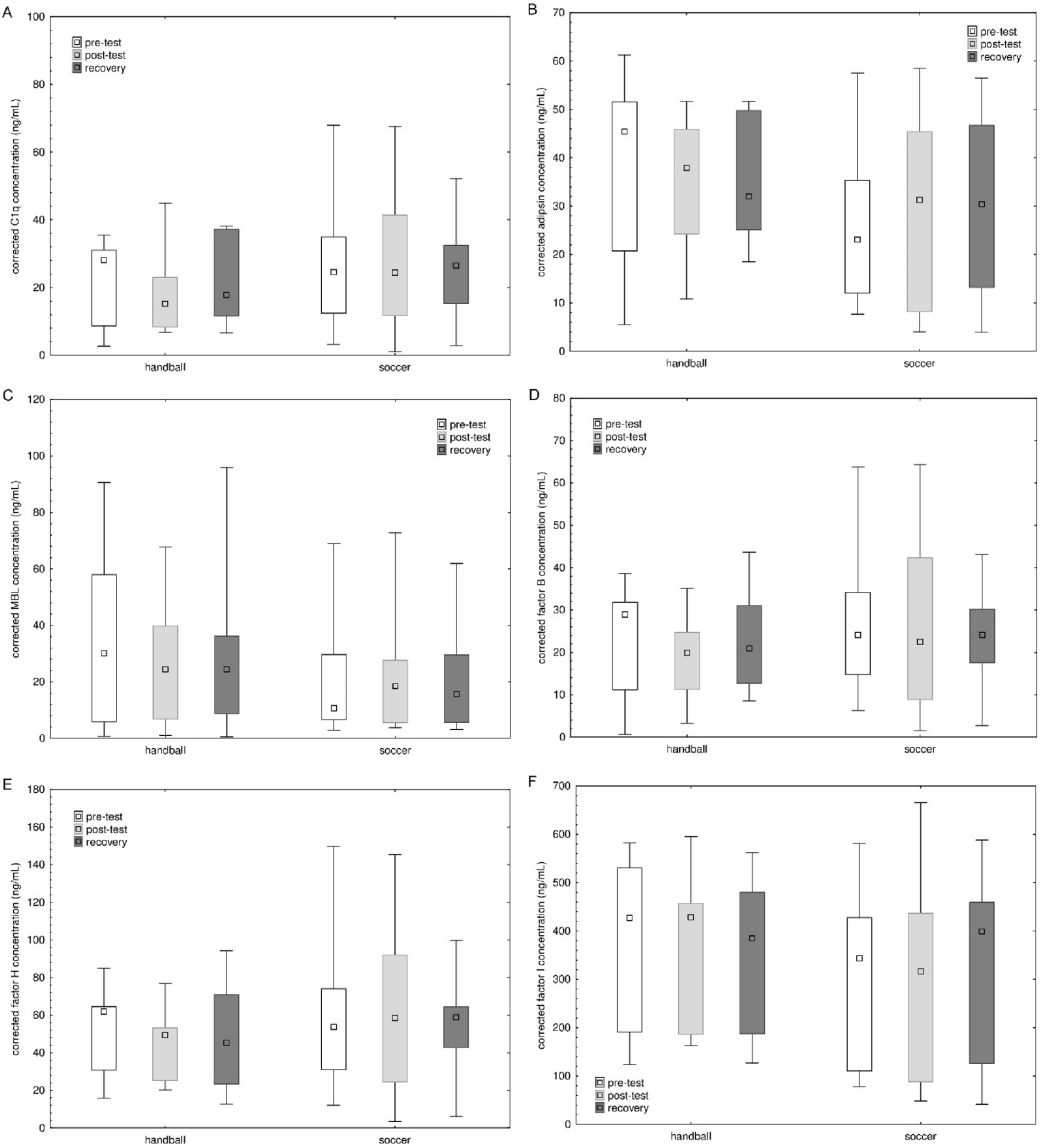
The plasma concentrations of regulatory CS compounds: A) C1q, B) adipsin, C) MBL, D) factor B, E) factor H, F) factor I.

Similarly, no significant changes were found in the studied activator components’ proteins (C1q, adipsin, MBL, factors B, H, and I) following physical exercise compared to baseline values (Fig 4).

The same results were noticed during the recovery time point in the blood plasma of handball and soccer players (Figs 2-4).

The level of inflammatory markers (C3/C4 plasma ratio) in handball players was equal to 0.09 (0.08–0.09) in the pre-test and was not changed in post-effort (0.09; 0.9–0.11) and recovery time points (equal to 0.08; 0.07–0.09). Conversely, in soccer players, the ratio was 0.15 (0.15–0.19) before the endurance effort on a mechanical treadmill, 0.16 (0.13–0.31) after a 24-hour recovery, and 0.22 (0.05–0.20) at the post-effort time point. These observations are consistent with changes in CRP concentration.

The data that support the abovementioned findings of the study are presented in the S1 Table.

## Discussion

Within a population, variability in the expression levels of complement proteins and other components leads to differences in steady-state complement activity among healthy individuals [29]. From the literature data, the normal range of C3 concentrations is usually between 80 and 178 ng/mL, and the reference range of C4 in serum is 10–40 ng/mL (100–400 mg/L) [11,30]. Data from the study by Gaya da Costa et al. 2018: the median C1q was 141 mg/L in men and 136 mg/L in women [11], while the physiological concentration of Factor H falls within the range of 0.116–0.562 mg/ml [11,30]. According to the study by Gaya da Costa et al., women have levels that are 53% lower (C5), 15% lower (C7), 59% lower (C8), and 14% lower (C9) than men. Concentrations of C8 above 50 µg/ml were observed exclusively in men (65% of the men studied had C8 concentrations above 50 µg/ml). Factor D concentrations were significantly higher in women (140%, interquartile range, IQR 115%–200%) than in men (100%, IQR 82%–137%), while the median MBL concentrations were 533 ng/mL (142–1076) in women and 843 ng/mL (289–1646) in men. The range is very wide due to genetic polymorphisms of the MBL2 gene [11], and these data indicate that the concentration values obtained in the studied groups of women fall within the ranges reported for young Caucasian women. It should also be noted that reference value ranges are dependent on the analytical methods used to measure the concentrations of the above-mentioned proteins.

The primary finding of this study is that progressive endurance effort until exhaustion on a mechanical treadmill did not trigger activation of the complement system in well-trained female athletes, as no significant changes in the level of C3, C4, C1 esterase, C2, C5, C4b, C5a, C3b/iC3b, adipsin, MBL, or factors B and H were observed at either the immediate post-effort or recovery time points. To account for the potential confounding effect of dehydration, the Dill and Costill equation [27] was applied to correct the results for plasma volume loss in all concentration calculations. After this correction, no significant changes remained. These results are consistent with findings by Semple et al., who described no post-effort changes in C3, C4, or C1 esterase among well-trained marathon athletes [31], and with the observation that no significant alterations in those proteins occurred following a marathon run among well-trained women [31]. The lack of post-effort changes in regulatory proteins- including C1q, adipsin, MBL, and factors B, H, and I – further supports the conclusion that the incremental exercise protocol did not engage the complement cascade in this population. To better understand the characteristics of the effort performed by both studied groups, it must be added that differences in final post-effort LA concentration as a secondary criterion used to confirm that a maximal effort was achieved during the running test found in soccer and handball players are in line with literature data [32–34]. In both female soccer and handball players, peak lactate concentrations after a maximal endurance test to fatigue on a mechanical treadmill range from 8 to 12 mmol·L^-1^, while female handball players are more likely to reach the upper end of these ranges due to the greater proportion of anaerobic effort in their game profile (throwing, jumping, contact tackling). Female soccer players may show slightly lower peak lactate values during laboratory testing, in part due to differences in muscle fiber recruitment relative to game-specific effort. Both groups, however, demonstrate lower lactate concentrations, typical of women compared with men, in the same sports [34]. Raeder et al. described that in elite female soccer players, a relatively low metabolic effort is observed during multidirectional sprint protocols, reflected by blood lactate concentration values ranging from 3.12 to 4.52 mmol/L, indicating a lower contribution of anaerobic glycolysis and suggesting a metabolic equilibrium close to the steady-state lactate level [35]. According to the study by Gabrys et al., blood lactate concentration in handball players after a run to exhaustion test (ramp test) is 10–12 mmol/L [33]. It is worth noting that some athletes generate high initial lactate values but also achieve higher maximum values at full load, compared to athletes who tend to have more slow-twitch muscle fibers [32–34].

These findings contrast with several studies that did observe exercise-induced alterations in the complement system. Karacabey et al. reported a statistically significant reduction in C3 and C4 immediately after both aerobic (30 minutes of treadmill running at approximately 60%−70% of cardiac reserve) and anaerobic (Wingate test) exercise in elite female volleyball players [36]. Chishaki et al., studying 25 highly trained female judokas, observed a significant increase in C3 in the subgroup with minor plasma volume loss following a judo practice session, and found a statistically significant positive correlation between dehydration state and the change ratios of C3 and C4 [37]. In contrast, Yaegaki et al. found no significant changes in C3 and C4 in female judokas studied 20 days before competition, regardless of whether they underwent weight reduction [38]. Taken together, these data suggest that complement response to exercise is heterogeneous and sensitive to the degree of plasma volume shift, underscoring the importance of the plasma volume correction applied in the presented study.

Studies involving lighter or moderate exercise protocols also yield variable results. In healthy women, 8 weeks of treadmill training at 60% VO_2_max (three times per week) resulted in significant reductions in post-exercise levels of C1q, C3, and factor H [8]. In a study of postmenopausal women, increased sedentary behavior was associated with higher C3 concentrations, while greater light physical activity was associated with lower C3 concentrations [39]. Ramanjaneya et al. found that the same moderate aerobic training protocol significantly reduced C1q. C3, and factor H in a health control group, but produced no changes in participants with polycystic ovary syndrome (PCOS). Moreover, C3, C4, factors B and H, properdin, and C4b differed significantly between groups following exercise [8]. These findings collectively indicate that the complement system responds differently across participant groups and exercise modalities, which may partly explain why no activation was detected in the present cohort of well-trained athletes.

Changes in individual complement components following high-intensity or prolonged effort have been documented in other contexts. Elevated levels of cleaved C5 (C5a) have been reported in the plasma of male marathon runners [40,41], and C6 was significantly elevated at 24 and 72 hours post-marathon among male participants [31]. Regarding the regulatory protein, factor B was significantly elevated 72 hours after marathon completion, though it did not exceed reference values [31]. Additionally, Ytting et al. found no influence of age, gender, menstrual cycle, or submaximal cycling exercise (25 minutes, at 70%−80% of expected HRmax) on MBL and MASP-2 protein levels, which are involved in lectin pathway activation and inhibition, respectively [42]. Elevated C1q plasma levels have been associated with muscle fibrosis, decreased muscle mass, and a negative correlation with thigh cross-sectional area and muscle strength [43]. This observation may be relevant to understanding the sex-related differences in complement response discussed below.

The absence of complement activation in the present study is consistent with findings from other female cohorts and contrasts with results previously reported in young physically active males performing the same incremental exercise test under comparable conditions. Because the present study did not include a male group for comparison, direct conclusions about sex-related differences cannot be drawn from these data alone. Nevertheless, when interpreted alongside existing literature and our previous study, the pattern is suggestive: the post-effort complement response might differ between sexes. Several biological mechanisms could contribute to such a difference. Rapid hormonal changes associated with the ovulatory cycle influence interferon gamma (IFN-γ) levels, B cell activity, and antibody production, and estrogen upregulates the Th1 cell response at lower concentrations and the Th2 cell response at higher concentrations [44] X-linked factors may also play a role, as overexpression of specific X-linked genes is associated with more severe forms of autoimmune diseases, such as systemic lupus erythematosus, in which the complement system is strongly involved [45]. However, detailed comparative studies involving great numbers of participants are needed to determine whether these mechanisms explain the lack of complement activation following exercise in women.

## Conclusions

One of the evolutionarily oldest and essential parts of the immune system is CS, which is involved not only in the pathogen-related immune response but also in the immunomodulatory effect related to increases in damage-associated molecular patterns and tissue regeneration. The lack of CS activation observed in the studied group of young women suggests that the endurance effort until exhaustion on the mechanical treadmill did not activate innate immunity via this pathway, and that any exercise-mediated immunomodulatory effect in this population was not accompanied by a complement-mediated inflammatory response. This observation is consistent with the hypothesis that endurance exercises in a young, physically active female population exert immunomodulatory rather than pro-inflammatory effects, particularly at the beginning of preparatory phases of the competition season, which could help in more efficiently utilizing knowledge of regenerative processes involving the immune system to build physiological condition across the season.

Given the study’s relatively small sample size and the absence of a male comparison group, the hypothesis that the lack of CS involvement in post-incremental exercise immunomodulation represents a sex-related immunological difference requires future evaluation in dedicated comparative studies.

## Supporting information

S1 Table The data that support the findings of the study. (XLSX)
